# Evolutionary history, longevity and terrestriality predict *Toxoplasma gondii* seroprevalence in free-ranging non-human primates

**DOI:** 10.1016/j.ijppaw.2025.101143

**Published:** 2025-09-30

**Authors:** Fidisoa Rasambainarivo, Billy Hinson, Olivier Rasolofoniaina, Sara Chelaghma, Randall E. Junge, C. Jessica E. Metcalf, Cathy V. Williams, Benjamin Rice

**Affiliations:** a Department of Biology, East Carolina University, Greenville, NC, USA; b Mahaliana Labs, Antananarivo, Madagascar; c Department of Biochemistry, University of Cambridge, Cambridge, UK; d Columbus Zoo and Aquarium, Columbus, OH, USA; e Department of Ecology and Evolutionary Biology, Princeton University, Princeton, NJ, USA; f Duke Lemur Center, Durham, NC, USA

## Abstract

Evidence from captive populations indicates that lemurs are particularly vulnerable to toxoplasmosis, a parasitic disease caused by *Toxoplasma gondii.* In wild populations, however, seroprevalence in lemurs remains low. This may be partly due to their predominantly arboreal behavior, which limits exposure to environmentally transmitted oocysts. Alternatively, or additionally, low seroprevalence could reflect high mortality following infection due to limited evolutionary exposure to the parasite and, consequently, a lack of evolved resistance. In this study, we assess whether the evolutionary history of primates with felids influences susceptibility to *T. gondii* infection, independent of ecological exposure. Specifically, we predicted that (1) species with greater terrestriality would exhibit higher exposure risk, (2) species longevity would be positively associated with their seroprevalence to *T. gondii* and (3) primate superfamilies with longer histories of co-occurrence with felids would show higher seroprevalence than Lemuroidea at similar levels of terrestriality and longevity. Serum samples from 435 free-ranging lemurs were tested for *T. gondii* antibodies and a literature review of *T. gondii* seroprevalence in free-ranging primates was conducted. The overall seroprevalence in Lemuroidea was 5.4 %, significantly lower than that observed in Ceboidea (11.8 %) and Cercopithecoidea (27.6 %). Notably, seroprevalence in lemurs was lower than expected based on their terrestriality, suggesting that evolutionary isolation from felids may underlie heightened vulnerability. Longevity modifies the risk profile in a lineage-specific way where seroprevalence increases with lifespan in Cercopithecoidea but not for lemurs. Collectively, our findings support the hypothesis that lemurs are immunologically naïve to *T. gondii*, and in the face of expanding domestic cat populations and increasing habitat fragmentation, the parasite may pose an underrecognized conservation threat.

## Highlights

• Lemurs have lower T. gondii seroprevalence (5.4%) than Ceboidea (11.8%) and Cercopithecoidea (27.6%).
• Low lemur seroprevalence cannot be explained by arboreality alone, implying other factors beyond ecological exposure.
• Evolutionary isolation from felids constrained immune adaptation, increasing lemur vulnerability.
• Lower-than-expected prevalence in lemurs suggests evolutionary history shapes susceptibility beyond ecology alone.
• Habitat fragmentation and cat introduction likely amplify lemur exposure to T. gondii, exacerbating conservationrisks.

## Graphical abstract

**Figure:**
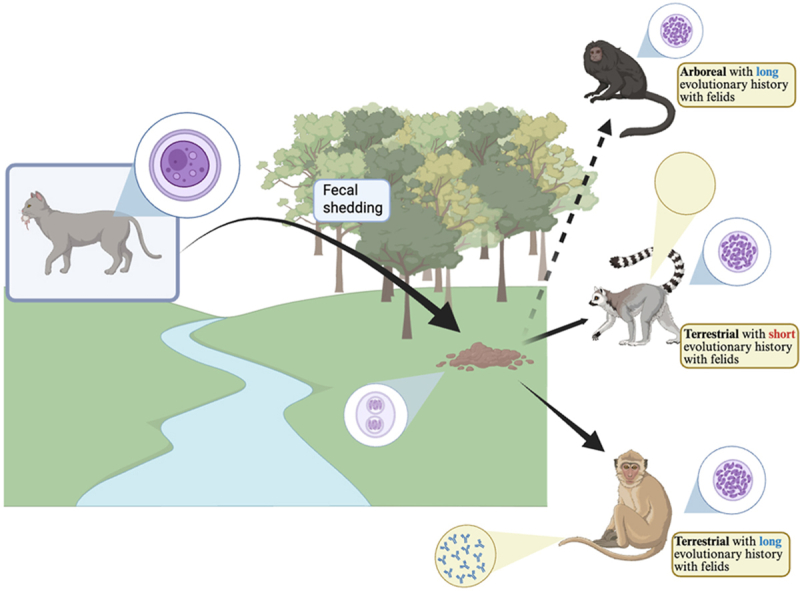

## 1 Introduction

The introduction of novel parasites to naïve hosts, populations, or species poses a growing threat to endangered animals, plants, and ecosystems (McCallum, 2012; Smith et al., 2006). Anthropogenic pressures, including habitat change and the movement of species, have heightened the risk of exposure to novel pathogens, thereby compounding existing threats to global biodiversity and conservation efforts (Baker et al., 2021; Daszak et al., 2001).

**Fig. 1:**
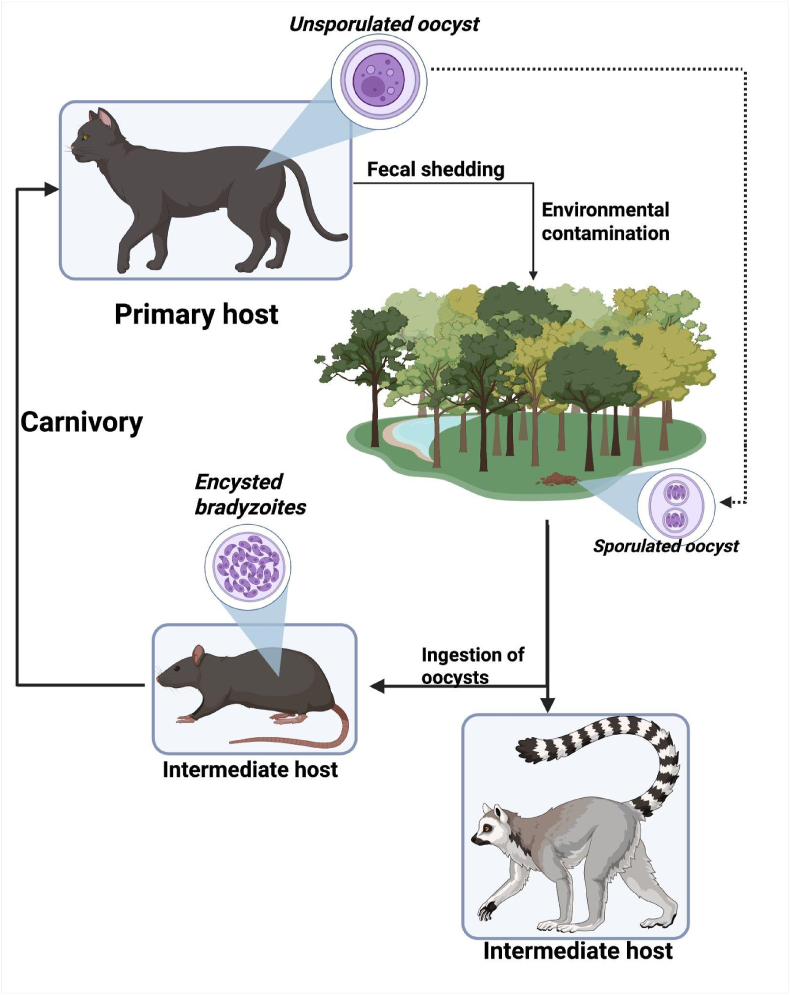
Life cycle of *Toxoplasma gondii*: Created in BioRender. https://BioRender.com/62z5vcm.

*Toxoplasma gondii* is a globally distributed protozoan parasite capable of infecting virtually all warm-blooded animals including humans, mammals, and birds. The only known definitive hosts of *T. gondii* are members of the Felidae family (domestic and wild cats) (Tenter et al., 2000). Infected cats may shed millions of environmentally resistant oocysts in their feces (Frenkel, 1988, Miller et al., 2023, Hutchison, 1965, Dubey et al., 1970, Frenkel et al., 1975). These oocysts can remain viable in soil or water for months under favorable climatic conditions (Dumètre and Dardé, 2003). Transmission occurs via ingestion of oocysts through contaminated food or water, vertical transmission from mother to fetus, or consumption of cyst-infected tissues in carnivorous species (Fig. 1) (Frenkel, 1988).

In most hosts, toxoplasmosis results in chronic, asymptomatic infection (Hill et al., 2005). However, in immunocompromised individuals or highly susceptible species, it can cause severe and potentially fatal disease (Dubey, 2022; Hill et al., 2005). Clinical manifestations include anorexia, lethargy, blindness, respiratory distress, neurological symptoms and sudden death. Sublethal infections may also impair fitness through reproductive complications, such as abortion, stillbirth, and neonatal mortality (Aguirre et al., 2019; Hill et al., 2005). Notably, toxoplasmosis has been identified as a significant cause of mortality in endangered species, including the southern sea otter (*Enhydra lutra*), ‘Alalā (*Corvus hawaiiensis*), and the golden-headed lion tamarin (*Leontopithecus chrysomelas*), thereby complicating conservation initiatives (Dietz et al., 1997; Miller et al., 2023; Work et al., 2000).

Marine mammals, Australian macropods, and Malagasy lemurs are highly susceptible to *T. gondii*, a pattern hypothesized to reflect their evolutionary history in environments devoid of felids and therefore of *T. gondii* exposure (Dubey, 2022; Dubey et al., 2020, 2021). Toll-like receptors (TLRs), a family of pattern recognition receptors involved in the innate immune detection of *T. gondii*, show markedly different evolutionary trajectories across mammalian lineages, shaped by their shared evolutionary histories (Gazzinelli et al., 2014). This variation suggests that host species lacking a co-evolutionary history with *T. gondii* or related apicomplexan parasites may possess less effective innate immune sensors. Consequently, these hosts may be more vulnerable to infection due to a diminished capacity to rapidly detect the parasite, which may in turn impair the activation of downstream adaptive immune responses (Gazzinelli et al., 2014).

While there are numerous case reports of fatal toxoplasmosis in captive neotropical primates and lemurs, studies reporting seroprevalence in free-ranging populations remain limited and typically describe low levels of antibody detection (Catão-Dias et al., 2013; Dubey, 2022; Epiphanio et al., 2003; Spencer et al., 2004). This pattern may reflect low environmental exposure, potentially due to an arboreal lifestyle that limits contact with contaminated soil. Among extant primates, terrestriality is common in members of the Cercopithecoidea (Old World monkeys) and Hominoidea (apes), but remains rare in the Ceboidea (neotropical monkeys), Lemuroidea (lemurs), Lorisidae (lorises and galagos), and Tarsiidae (tarsiers) (Eppley et al., 2022; Estrada and Marshall, 2024). Species exhibiting greater degrees of terrestrial behavior are therefore expected to have increased exposure risk to environmentally transmitted pathogens such as *T. gondii*. Additionally, as *T. gondii* prevalence tends to increase with individual age, longer-lived primate species may exhibit higher overall prevalence due to the cumulative risk of exposure over time (Dámek et al., 2023).

Alternatively, the low seroprevalence in free ranging primates may suggest high pathogen virulence and low host tolerance, leading to rapid mortality in infected individuals and thereby reducing the likelihood of detecting antibodies in population-level surveillance (Obendorf et al., 1996). Conflicting scenarios have been proposed to explain the evolutionary history and subsequent global spread of *T. gondii.* Some studies suggest that the parasite originated in South America approximately 1.5 million years ago, while others propose an origin in Africa/Asia, with South America subsequently becoming a hotspot of diversification (Bertranpetit et al., 2017; Galal et al., 2019, Galal et al., 2022). Regardless of the scenario, both imply that Old World and New World nonhuman primates have had a longer evolutionary history with the parasite compared to Malagasy primates. In contrast, felids, and therefore *T. gondii*, were introduced to Madagascar relatively recently, roughly 1000 years ago through human migration (Sauther et al., 2020). This disparity in host–pathogen coevolution presents a unique opportunity to examine how evolutionary history influences susceptibility to and prevalence of *T. gondii* across primate taxa.

This study investigates whether evolutionary interactions with felids reflect differences in *T. gondii* seroprevalence among primates, beyond environmental exposure alone. High seroprevalence may indicate both significant exposure and survival post-infection, whereas low or absent seroprevalence may reflect either minimal exposure or high mortality following infection.

Our objectives were: (1) to estimate *T. gondii* seroprevalence in free-ranging lemurs, and (2) to assess the relationship between terrestriality, longevity, evolutionary history and seroprevalence across primate species. We hypothesize that longer lived species and species exhibiting greater terrestriality will show higher exposure rates. Additionally, we predict that members of the Hominoidea, Ceboidea and Cercopithecoidea, primate superfamilies with a longer evolutionary history alongside felids and *T. gondii*, will exhibit higher seroprevalence than Lemuroidea, even at similar levels of terrestriality.

## 2 Methods

### 2.1 Lemur sample serum collection

Serum collection and health evaluations of free ranging lemurs were conducted using methods consistent with previously published protocols for free-ranging lemur health assessments (Junge et al., 2017; Page-Karjian et al., 2021; Rasambainarivo et al., 2014). Briefly, lemurs were individually anesthetized using intramuscular tiletamine and zolazepam (Telazol, Fort Dodge Animal Health, OverlandPark, KS 66225, USA; 10 mg/kg, dose based on estimated body weight), administered by remote injection (Type ‘‘P’’ Disposable Dart, Pneu-Dart, Williamsport, PA 17701, USA). Each anesthetized lemur was weighed. The sex and age category of the animal were recorded. Animals were classified as immature or adult based on body size, dentition, and allometric measurements. A subcutaneous radio transponder chip (Trovan Ltd., Weilerswist 53919, Germany) was implanted in the interscapular region of the upper back of each lemur for permanent identification. Blood samples not exceeding 1 % of body weight were collected from each animal. Activities in this project complied with all United States standards for the use of animals in research and the protocol was approved by Saint Louis Zoo, Columbus Zoo and Aquarium, Omaha Henry Dorly Zoo (97-001, 12–101) and Duke University (A028-14-02) Animal Use Committee. Methods conformed to the guidelines of the American Society of Mammalogists (Sikes and Mammalogists, 2016) and adhered to all research requirements in Madagascar (permits number 63/17; 70/17; 41/18; 322/19; 045/21).

Blood was placed into non-anticoagulant tubes and allowed to clot. Tubes were centrifuged within 8h of sample collection. Serum samples were frozen in liquid nitrogen for transport and stored at −80C until analysis.

Serum samples collected between 2004 and 2012 were submitted to the University of Tennessee Comparative Parasitology Service (Knoxville, TN) and analyzed for *T. gondii* immunoglobulin G (IgG) using Enzyme Linked ImmunoSorbent Assay (ELISA). Given its simplicity, cost-effectiveness, and applicability across host species, the modified agglutination test (MAT) was used to assess the seroprevalence of *T. gondii* in lemurs sampled between 2013 and 2024. The commercially available *Toxoplasma* Whole-cell Antigen for MAT (TgMAT, University of Tennessee Research Foundation, Memphis, TN, USA) was used according to the manufacturer's instructions. A cutoff titer of >1:25 was used to classify samples as seropositive, following recommended protocols. Samples were tested in triplicate.

### 2.2 Literature search for *T. gondii* seroprevalence in primates

We conducted a systematic literature review to identify studies reporting *T. gondii* seroprevalence in free-ranging primates. Six electronic databases (Scopus, PubMed, Web of Science, ScienceDirect, ProQuest, and Google Scholar) were searched for studies published between January 1990 and March 2025. The search strategy used combinations of the following terms: (“*Toxoplasma gondii*” OR “*T. gondii*” OR “*Toxoplasma* infection” OR “Toxoplasmosis”) AND (“prevalence” OR “seroprevalence” OR “epidemiology”) AND (“primate”). We also manually screened the reference lists and citing articles of all selected studies to identify additional relevant sources.

Studies were included based on the following criteria: (1) full-text or abstract available in English, (2) peer-reviewed original research articles, short reports, or letters to the editor examining *T. gondii* prevalence in free-ranging primates (3) publication date between January 1990 and March 2025 with a digital object identifier (DOI), and (4) clear reporting of sample size and number of positive cases. Exclusion criteria included studies focusing on captive populations (e.g., zoos, sanctuaries), studies on human seroprevalence, research relying solely on PCR detection methods, and datasets previously published by authors of the present study to avoid duplication.

#### 2.2.1 Analysis of species-specific factors: terrestriality and lifespan

To quantify terrestriality, we used datasets published by Eppley et al. (2022) and Estrada and Marshall (2024), which provide quantitative estimates of terrestrial behavior in 515 extant nonhuman primate species (Eppley et al., 2022; Estrada and Marshall, 2024). When available, the percentage of active time spent on the ground was used to classify primates’ terrestriality.

To assess the association of *T. gondii* seroprevalence with species longevity as a proxy for long-term infection risk, we extracted maximum recorded lifespan values from the AnAge 2.2.1 database, a curated resource of life history and longevity traits across vertebrates (Magalhães et al., 2023; Magalhães and Costa, 2009). For both terrestriality and longevity, genus-level data were used when species-specific values were not available.

#### 2.2.2 Statistical analysis

We used a series of generalized linear models (GLMs) with a Gaussian error distribution to investigate the ecological and evolutionary predictors of *T. gondii* seroprevalence across nonhuman primate species. The response variable was species-level seroprevalence, calculated as the proportion of seropositive individuals per species. Predictor variables included mean terrestriality (treated as a continuous, mean-centered variable and scaled), longevity (continuous, mean-centered and scaled) and primate superfamily (categorical: Ceboidea, Cercopithecoidea, Hominoidea, and Lemuroidea). We included two-way interactions between superfamily and longevity, and between superfamily and terrestriality, to test whether the effect of longevity or terrestriality on *T. gondii* seroprevalence varies across primate superfamilies.

We performed model selection based on Akaike Information Criterion corrected for small sample sizes (AICc). Competing models were ranked by their AICc values, and models within ΔAICc ≤2 were considered to have comparable support.

## 3 Results

### 3.1 *T. gondii* seroprevalence in wild lemurs of Madagascar

Serum samples from 435 adult lemurs of 21 species were collected between 2004 and 2024 and tested for the presence of antibodies (IgG) against *T. gondii*. Of those, 16 (3.7 %) individuals from 6 species tested positive at titers 25 or above (Table 1).

**Table 1:** Methods and results of *Toxoplasma gondii* serological testing in 435 individuals from 20 lemur species sampled across Madagascar.

Species	n tested	Toxoplasma positive	Serological method
*Avahi* sp	37	2	MAT
*Daubentonia madagascariensis*	1	0	ELISA
*Eulemur albifrons*	15	2	ELISA
*Eulemur coronatus*	4	0	ELISA
*Eulemur fulvus*	5	0	ELISA
*Eulemur macaco*	10	1	ELISA
*Eulemur rubriventer*	2	0	ELISA
*Eulemur sanfordi*	7	0	ELISA
*Hapalemur griseus*	2	0	ELISA
*Indri indri*	74	0	MAT
*Lemur catta*	21	0	ELISA
*Lepilemur mustelinus*	13	0	ELISA
*Prolemur simus*	21	0	ELISA
*Propithecus candidus*	1	0	ELISA
*Propithecus deckeni*	18	1	ELISA
*Propithecus diadema*	125	8	MAT
*Propithecus perrieri*	2	0	ELISA
*Propithecus tattersalli*	9	1	ELISA
*Propithecus verreauxi*	23	0	ELISA
*Varecia rubra*	14	0	ELISA
*Varecia variegata*	31	1	ELISA

### 3.2 Literature search for *T. gondii* seroprevalence in lemurs and neotropical primates

**Fig. 2:**
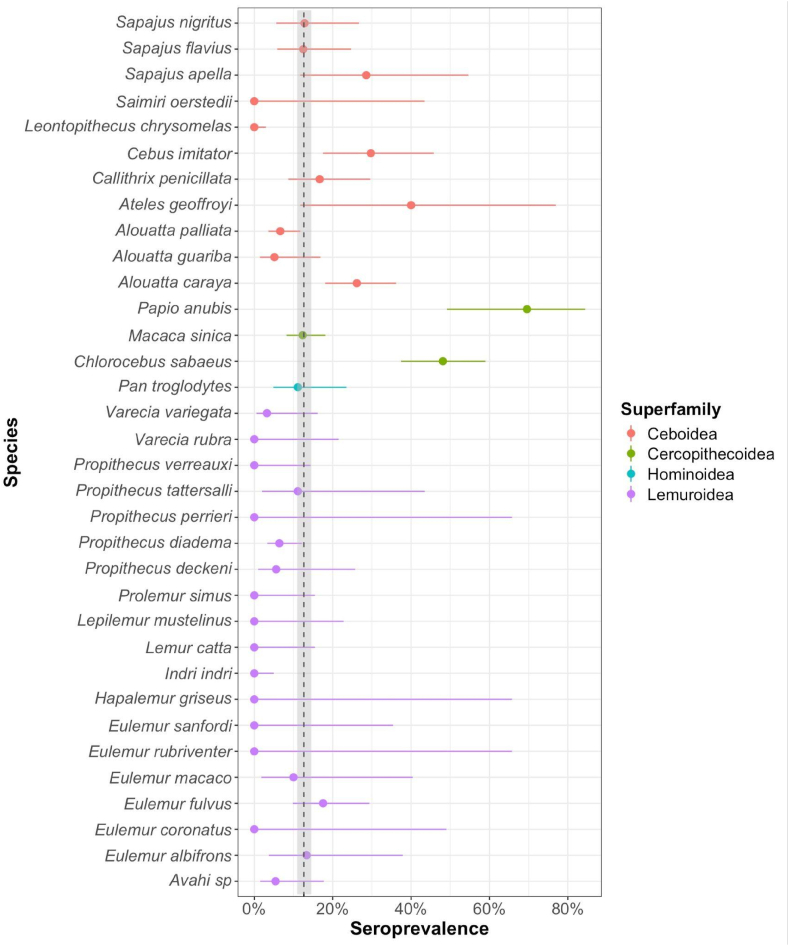
Forest plot of seroprevalence to *Toxoplasma gondii* in primate species with means shown by closed circles and whiskers representing the 95 % confidence interval. Dashed line and shaded area represent mean global *T. gondii* seroprevalence and 95 % Confidence interval in free-ranging non-human primates.

The literature search initially identified 281 articles. After removing duplicates and excluding studies that did not meet the eligibility criteria, 13 articles were retained, reporting data from 969 non-human primate samples representing 16 species (supplementary material S1). Among these, one study reported *T. gondii* seropositivity in 10 out of 52 brown lemurs (*Eulemur fulvus*) tested on Mayotte (Quintard et al., 2019). The inclusion of the Mayotte lemur data broadens both taxonomic and geographic coverage within Lemuroidea, contributing to a more comprehensive assessment of *T. gondii* exposure across the clade. Although these populations are introduced, they are free-ranging, and thus provide ecologically relevant insights into exposure under natural foraging and social behaviors. When combined with the prevalence estimates from wild lemurs presented in Section 3.1, the overall *T. gondii* seroprevalence is 5.3 % (95 % CI: 3.58–7.83 %) in Lemuroidea (n = 487), 11.8 % (95 % CI: 9.4–14.37 %) in Ceboidea (n = 600), 27.6 % (95 % CI: 22.43–33.36 %) in Cercopithecoidea (n = 272) and 11.11 % (95 % CI: 4.16–24.84 %) in Hominoidea (n = 45) (Fig. 2). Combined with the current study, seroprevalence data now encompass 37 of the 505 recognized primate taxa (7.3 %), with an overall global seroprevalence estimate of 12.62 % (95 % CI: 10.95–14.5 %).

### 3.3 Influence of terrestriality and longevity and evolutionary history on exposure to *T. gondii* in primates

We fitted a generalized linear model to evaluate the influence of terrestriality, evolutionary history (superfamily), and lifespan (maximum longevity) on *T. gondii* seroprevalence among non-human primates. The best-supported model includes two-way interactions between superfamily and longevity and between superfamily and terrestriality (Table 2).

**Table 2:** Model selection results for *T. gondii* exposure in primates.

Model Formula	df	AICc	ΔAICc	w
Terrestriality ∗ Superfamily + Superfamily ∗ Longevity	10	−65.58	–	0.99
Terrestriality ∗ Superfamily + Longevity	8	−56.26	9.32	0.01
Terrestriality + Superfamily + Longevity	6	−48.15	17.43	<0.001

**Fig. 3:**
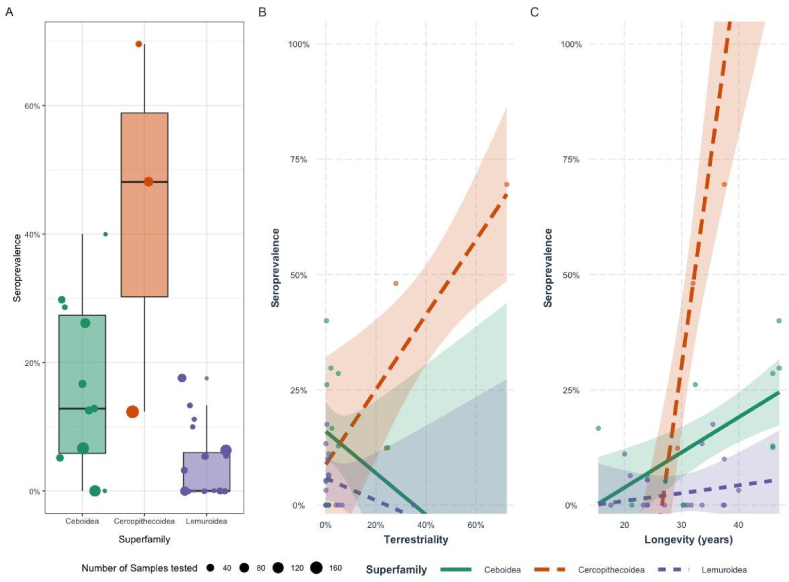
Seroprevalence of *Toxoplasma gondii* in superfamilies of free ranging non-human primates (3-A). Seroprevalence (±CI 95 %) of T. gondii in free ranging non-human primates obtained from selected generalized linear models (GLM) as a function of their terrestriality (3-B), and longevity (3-C).

Compared to Ceboidea, members of the Cercopithecoidea superfamily exhibited significantly higher baseline seroprevalence (β = 0.568, p < 0.001), whereas Lemuroidea had significantly lower values (β = −0.10, p = 0.005; Fig. 3A). Across primates, terrestriality was not significantly associated with seroprevalence (β = −0.09, p = 0.10). However, this effect varied by superfamily: in lemurs, terrestriality was not a strong predictor (β = −0.68, p = 0.31), while in Cercopithecoidea it was positively and significantly associated with exposure (β = 0.19, p = 0.014; Fig. 3B). Longevity was positively associated with seroprevalence overall (β = 0.07, p = 0.001). Again, the effect differed by lineage: in Cercopithecoidea, increasing longevity was strongly associated with higher seroprevalence (β = 0.718, p = 0.001), whereas in lemurs, longevity was not predictive of exposure (β = 0.053, p = 0.097; Fig. 3B).

## 4 Discussion

Our study aimed to estimate the prevalence of *Toxoplasma gondii* exposure in free-ranging lemurs and place these data within a broader comparative framework across primate superfamilies. This framework draws on a meta-analysis of studies employing different serological assays (e.g., MAT, ELISA, IFAT) and positivity thresholds, introducing variability in exposure estimates (Huertas-López et al., 2024). Although such heterogeneity may influence prevalence values in some species, the consistent patterns we observed across superfamilies and life-history traits suggest that our comparative conclusions are robust. Standardized diagnostic protocols would further enhance the precision of cross-species comparisons. Within this context, our findings demonstrate that evolutionary history with felids (superfamily) interacts in complex ways with behavior (terrestriality) and life-history traits (longevity) to shape seroprevalence patterns.

Members of the superfamily Lemuroidea were significantly less likely to exhibit antibodies to *T. gondii* compared to other primate superfamilies. This pattern suggests either reduced exposure to the parasite or high post-infection mortality, both of which have important implications for understanding host–pathogen dynamics. Low seroprevalence in lemurs is particularly striking given the widespread presence of domestic and feral cats across Madagascar, including in primary forests and protected areas, which provides ample opportunity for oocyst exposure (Farris et al., 2015; Rasambainarivo et al., 2017; Sauther et al., 2020). One plausible route of exposure is geophagy, the voluntary ingestion of soil, which is common among primates and can provide physiological benefits such as mineral supplementation and detoxification (Krishnamani and Mahaney, 2000; Miao et al., 2015; Semel et al., 2019). However, geophagy also carries parasitological risks, including exposure to soil-borne pathogens like *T. gondii* and *Trichuris* (Deng et al., 2021; Pebsworth et al., 2012, 2019). In addition to soil ingestion and trophic transmission via invertebrates, waterborne exposure may represent a potential route of *T. gondii* infection in lemurs, particularly for individuals that drink from streams, ponds, or other surface water sources, where oocysts can persist in the environment (Shapiro et al., 2019). This route may be especially relevant in dry or arid habitats, where research suggests that feces avoidance is a secondary consideration in waterhole selection (Amoroso et al., 2019).

Alternatively, the low seroprevalence observed in lemurs may reflect high mortality following acute infection. While no systematic studies have documented causes of death in free-ranging lemurs, multiple reports of fatal toxoplasmosis in captive individuals (Denk et al., 2022; McAloose and Stalis, 2018; Spencer et al., 2004) support the hypothesis that wild populations could also experience high lethality upon exposure.

Because *T. gondii* exposure accumulates over time, longer-lived species are predicted to show higher seroprevalence (Dámek et al., 2023). In our dataset, longevity was positively associated with exposure in Cercopithecoidea but not in lemurs. This decoupling indicates that, within Lemuroidea, age does not increase the likelihood of detectable antibodies. Such a pattern may reflect limited environmental exposure regardless of lifespan or elevated post-infection mortality, distinguishing lemurs from other primates and highlighting their unique susceptibility to *T. gondii*. Direct post-mortem analyses of free-ranging lemurs are urgently needed to determine causes of death and clarify the role of acute toxoplasmosis in shaping observed seroprevalence patterns.

It is worth noting that mitigation strategies for *T. gondii* infection in wildlife are emerging. For example, experimental vaccination trials in squirrel monkeys (*Saimiri sciureus*) have shown protective immune responses and reduced severity of clinical toxoplasmosis (Ducournau et al., 2023), suggesting potential applicability to other susceptible species. In parallel, control measures targeting the parasite's definitive hosts (domestic and feral cats) may be useful. Although no vaccines are currently licensed for domestic or feral cats, experimental vaccination and population control programs have been proposed as potential strategies to reduce *T. gondii* infection in definitive hosts and thereby decrease environmental oocyst contamination (Innes et al., 2019; Marinović et al., 2020; Mateus-Pinilla et al., 2002; Rasambainarivo et al., 2024). An integrated strategy combining host- and environment-focused interventions may offer the most effective means to reduce *T. gondii* transmission in free-ranging primates, though further research is needed to assess the feasibility, effectiveness, and ecological consequences of these approaches.

Strain variation may also influence infection outcomes, as certain *T. gondii* genotypes are more pathogenic than others (Khan et al., 2009). Identifying circulating strains using microsatellites (MS) and/or Next Generation Sequencing (NGS) methods is therefore essential for assessing infection risk and potential disease severity in wild primate populations and facilitate meaningful comparisons with prior and ongoing global studies (Joeres et al., 2023; Uzelac and Djurković-Djaković, 2025).

Despite the wide geographic distribution of wild primates and the known conservation implications of toxoplasmosis, relatively few studies have examined *T. gondii* seroprevalence in these populations. This represents a critical gap, especially compared to other taxa (Buschang et al., 2025; Wilson et al., 2020; Zaki et al., 2024). The relative lack of attention to wild primates underscores the urgent need to prioritize this group in future surveillance and research to fully understand the ecological and conservation consequences of *T. gondii* exposure.

## 5 Conclusion

In conclusion, our study reveals that *T. gondii* exposure in non-human primates is shaped by a combination of ecological, evolutionary, and life-history factors. The particularly low seroprevalence in lemurs, despite likely exposure, highlights their potential vulnerability and suggests that pathogen spillover may pose an underappreciated threat. Future work should focus on identifying environmental reservoirs, characterizing circulating strains, and evaluating host susceptibility to inform conservation strategies. As human activity continues to alter ecosystems and increase contact between wildlife and domestic animals, understanding and mitigating zoonotic risks will be essential for preserving the health and resilience of primate populations worldwide.

## CRediT authorship contribution statement

**Fidisoa Rasambainarivo:** Writing – review & editing, Writing – original draft, Visualization, Validation, Supervision, Resources, Project administration, Methodology, Investigation, Funding acquisition, Formal analysis, Data curation, Conceptualization. **Billy Hinson:** Writing – review & editing, Writing – original draft, Visualization, Formal analysis, Data curation, Conceptualization. **Olivier Rasolofoniaina:** Writing – review & editing, Writing – original draft, Methodology, Investigation, Formal analysis. **Sara Chelaghma:** Writing – review & editing, Writing – original draft, Visualization, Investigation, Formal analysis, Conceptualization. **Randall E. Junge:** Writing – review & editing, Writing – original draft, Validation, Supervision, Resources, Project administration, Methodology, Investigation, Funding acquisition, Formal analysis, Data curation, Conceptualization. **C. Jessica E. Metcalf:** Writing – review & editing, Writing – original draft, Resources, Project administration, Methodology, Funding acquisition, Formal analysis, Data curation, Conceptualization. **Cathy V. Williams:** Writing – review & editing, Writing – original draft, Supervision, Resources, Project administration, Methodology, Investigation, Funding acquisition, Formal analysis, Data curation, Conceptualization. **Benjamin Rice:** Writing – review & editing, Writing – original draft, Supervision, Project administration, Methodology, Formal analysis, Data curation, Conceptualization.

## Conflicts of interest statement

The authors whose names are listed below certify that they have **NO conflicts of interest to disclose** and NO affiliations with or involvement in any organization or entity with any financial interest (such as honoraria; educational grants; participation in speakers’ bureaus; membership, employment, consultancies, stock ownership, or other equity interest; and expert testimony or patent-licensing arrangements), or non-financial interest (such as personal or professional relationships, affiliations, knowledge or beliefs) in the subject matter or materials discussed in this manuscript.
